# LIVED EXPERIENCES OF OLDER CHINESE IMMIGRANTS IN AFFORDABLE HOUSING DURING THE PANDEMIC: STRESS AND RESILIENCE

**DOI:** 10.1093/geroni/igac059.247

**Published:** 2022-12-20

**Authors:** Kexin Yu, Iris Chi, Fei Sun

## Abstract

Older immigrants in affordable housing have unique sets of challenges during the COVID-19 pandemic posed by limited English proficiency and resources. The aggregated living condition could increase their risk of being exposed to the contagious disease. This symposium reports empirical findings of the social Network of Immigrant Chinese older adults in affordable Housing Environment (NICHE) project, which focused on the influence of COVID-19 on underprivileged Chinese older immigrants. We conducted 27 semi-structured interviews with foreign-born older Chinese immigrants (mean age 78.1, 69.23% female) in an affordable housing in Los Angeles to learn about their lived experiences and coping strategies during the COVID-19 pandemic. The first presentation focused on the changes in older Chinese immigrants’ social life after the onset of the pandemic and aimed to understand the pandemic’s impact on their depressive symptoms and loneliness. The second presentation describes older Chinese immigrants’ perceived pandemic-related stressors and resilience across phases of the pandemic, including at the beginning, after they got vaccinated, and the rising of delta variant. The participants explained what supportive services had been helpful and what support they wished they could have over the past two years in the pandemic. The third presentation reports the experience of being discriminated against during the pandemic, the Chinese older adults’ attitudes towards these discriminatory events, and coping strategies. Together, these three presentations will depict the lived experience of Chinese immigrants over two years during the pandemic and discuss intervention strategies and policy considerations for preparing for future crisis like the COVID-19 pandemic.

